# Application of topical pharmacological agents at the site of peripheral nerve injury and methods used for evaluating the success of the regenerative process

**DOI:** 10.1186/s13018-014-0094-3

**Published:** 2014-10-11

**Authors:** Agon Y Mekaj, Arsim A Morina, Cen I Bytyqi, Ymer H Mekaj, Shkelzen B Duci

**Affiliations:** Clinic of Neurosurgery, University Clinical Center of Kosovo, Rrethi i spitalit p.n., Prishtina, 10000 Kosovo; Clinic of Orthopaedic and Traumatology, University Clinical Center of Kosovo, Rrethi i spitalit p.n., Prishtina, 10000 Kosovo; Institute of Pathophysiology, Faculty of Medicine, University of Prishtina, Rrethi i spitalit p.n., Prishtina, 10000 Kosovo; Department of Plastic and Reconstructive Surgery, University Clinical Center of Kosovo, Rrethi i spitalit p.n., Prishtina, 10000 Kosovo

**Keywords:** Tacrolimus, Hyaluronic acid, Melatonin, Vitamin B12, Peripheral nerve injury

## Abstract

Traumatic injuries of the peripheral nerves are very common. Surgical repair of the damaged nerve is often complicated by scar tissue formation around the damaged nerve itself. The main objective of this study is to present the recent data from animal experimental studies where pharmacological topical agents are used at the site of peripheral nerve repair. Some of the most commonly topical agents used are tacrolimus (FK506), hyaluronic acid and its derivatives, and melatonin, whereas methylprednisolone and vitamin B12 have been used less. These studies have shown that the abovementioned substances have neuroprotective and neuroregenerative properties though different mechanisms. The successes of the regenerative process of the nerve repair in experimental research, using topical agents, can be evaluated using variety of methods such as morphological, electrophysiologic, and functional evaluation. However, most authors agree that despite good microsurgical repair and topical application of these substances, full regeneration and functional recovery of the nerve injured are almost never achieved.

## Introduction

The peripheral nervous system (PNS) is composed of the cranial nerves, which project from the brain and pass through foramina (openings) in the scull, and the spinal nerves, which project from the spinal cord and pass through intervertebral foramina of the vertebrae [1]. The PNS consists of motor and sensory neurons that are the largest and most spatially complex in the body. Peripheral nerve injuries are more frequent and may be accompanied by neurological deficits. In contrast to the central nervous system, the peripheral nervous system has competence to regenerate injured axon [2]. It is essential for clinicians to have an understanding of basic anatomy of PNS in order to classify and subsequently treat a nerve injury. Within the given peripheral nerve, fibers are organized in separate bundles known as fascicles. Less than half of the nerves are enclosed within myelin sheaths. The remaining unmyelinated fibers travel in deep gutters along the surface of Schwann cells [3]. Schwann cells are the key element for the promotion regeneration [4].

Regeneration of the damaged peripheral nerve depends on the microsurgical procedure performed. Currently, there are several operating techniques used to repair injured nerves such as direct epineural repair, grouped fascicular repair, fascicular repair, and nerve grafting. The results following nerve repairs are influenced by many parameters, such as the nature, location, and extent of the injury, the level and timing of the repair, the fascicular anatomy, appropriateness of re-alignment of the injured nerve, and the surgical technique, as well as patient factors [5].

In addition to these factors in the regeneration of nerve repair, some pharmaceutical agents which are used locally at the site of nerve repair also have an effect. Several studies have shown that the most frequently applied topical substances are tacrolimus (FK506), hyaluronic acid and its derivatives, melatonin, and methylprednisolone. These substances contribute to fibroblast proliferation suppression at the site of the peripheral nerve repair thus reducing scar formation in the injured peripheral nerve.

## Application of pharmacologic agents at the site of peripheral nerve repair

### Effects of topically administered FK506 on peripheral nerve regeneration

Tacrolimus (FK506) is a macrolide immunosuppressive drug that is approved for the prevention of allograft rejection. The abilities of tacrolimus are to modulate the immune system and to inhibit T cell function by binding to FK binding proteins (FKBP) and mediate immunosuppression by inhibiting calcineurin and calcium- and calmodulin-dependent phosphatase. The primary biologic effect of calcineurin inhibition includes the decrease of the production of inflammatory cytokines such as tumor necrosis factor (TNF)α, interleukin-2, and interferon-γ [6]. FK506 binds to its receptors. There are two types of receptors for FK506: FKBP12 and FKBP52. The FKBP12 receptors are responsible for immunosuppressive effects, whereas the FKBP52 receptors are related to neuroregenerative effects. Studies performed during the past decade have demonstrated FK506 in sub-immunosuppressive doses exhibits neuroprotective and neuroregenerative properties. This finding has stimulated interest in characterizing the neurophysiologic effect of FK506 with various nerve injury models [7]. FK506 has been proven to have neurotrophic actions in experimental models, increasing neurite elongation and accelerating the rate of nerve regeneration *in vitro* and *in vivo* [8]. It has been demonstrated that FK506, a generally applied immunosuppressant in organ transplantation, has a powerful effect of promoting axon regeneration through its immunosuppressive and neurotrophic action [9-11]. The topical effect of FK506 on peripheral nerve had not been well investigated to date. Results of the use of FK506 in peripheral nerve regeneration differ in the literature. One possible reason for the relative variability of the results of studies of experimental nerve injures is the variety of models and testing methods used [12]. FK506 could promote peripheral nerve regeneration through reducing scar formation; however, little is known about how FK506 reduces scar formation [13]. Prior studies have showed that FK506-FKBP12 interaction may lead neuroregenerative effect through increased neuronal expression of a growth cone-associated protein GAP-43, but there are evidence that this occurs through inactivation of neuronal nitric synthetase [14,15]. It is known that the key binding protein FKBP52 (also known as FKBP-59 or heat shock protein) is responsible for neurotrophic action of FK506. This (neurotrophic) action can be completely prevented by the addition of monoclonal antibody against FKBP52 *in vitro* [7] A reduction in scar formation at the site of nerve repair by the abovementioned mechanisms has been associated with better nerve function recovery.

### Effects of topically administered hyaluronic acid on peripheral nerve regeneration

Hyaluronic acid (HA) is a carbohydrate, more specifically a mucopolysaccharide, occurring naturally in all living organisms. It can be several thousands of sugars (carbohydrates) long. When not bound to other molecules, it binds to water giving it a stiff viscous quality similar to “Jell-o” [16]. Hyaluronic acid was discovered in bovine vitreous humor by Meyer and Palmer in 1934 [17]. It is most frequently referred to as HA due to the fact that it exists *in vivo* as a polyanion and in the protonated acid form [18]. The term “hyaluronan” was introduced in 1986 by Endre Balazs to conform with the international nomenclature of polysaccharides [19]. HA is an agent which is known to reduce the extent of scar formation by inhibiting lymphocyte migration, proliferation and chemotaxis granulocyte fagocitosis and degranulation, and macrophage motility [20]. The predominant mechanism of HA is unknown; *in vivo*, *in vitro*, and clinical studies demonstrate various physiological effects of exogenous HA. Of these effects, worth mentioning is its chondroprotective effects *in vitro* and *in vivo* (orthopedic application). HA has also been successfully used in ophthalmology, cardiovascular system, dermatology, and wound healing [16]. HA is a major component of the extracellular matrix, and it plays an important role in the early wound healing process [21]. HA is an endogenous stimulator of interleukin-1 (IL-1) production and IL-1 affects fibroblasts proliferation and collagenase production [22]. Exogenous HA enhances chondrocyte HA and proteoglycan synthesis, reduces the reproduction and activity of proinflammatory mediators and matrix metalloproteinases, and alters the behavior of immune cells. These functions are manifested in the scavenging of reactive oxygen-derived free radicals, the inhibition of immune complex adherence to polymorphonuclear cells, the inhibition of leukocyte and macrophage migration and aggregation, and the regulation of fibroblast proliferation [23]. HA and its derivatives may also promote regeneration of injured nerves through realignment of the fibrin matrix, and they can provide a suitable environment for axonal ingrowth. Use of the hyaluronic acid-carboxymethylcellulose (HA CMC) membrane Seprafilm as a solid anti-adhesion barrier agent is one of the therapeutic approaches to deduce postoperative scar formation and is effective in promoting peripheral nerve regeneration at the repair site [24,25]. HA gel significantly reduces nerve adhesions after type nerve injured [26]. Mohammad et al. describe the early effect on nerve regeneration of continuous local delivery of nerve growth factor (NGF) and the local incorporation of HA inside a newly manufactured nerve conduit material from fresh human amniotic membrane. Human amniotic membrane contains important biochemical factors that play a major neurotrophic role in the nerve regeneration process [27].

### Effects of topically administered melatonin on peripheral nerve regeneration

Melatonin (MLT) is the main hormone of the pineal gland. The pineal gland is in the middle of the brain, and it secretes MLT, a hormone that regulates when you sleep at night and wake up in the morning [28]. Melatonin has an effect on the morphologic features of the nerve tissue, suggesting its neuroprotective, free radicals scavenging, antioxidative, and analgesic effects in degenerative diseases of peripheral nerves. At present, it is widely accepted that melatonin has useful effect on axon length and sprouting after traumatic events to peripheral nerves [29,30]. As superoxide dismutase is an important antioxidative enzyme involved in redox regulation of regulative stress, melatonin would exert its beneficial effects by preserving the superoxide dismutase reactivity following peripheral nerve injury [31]. Melatonin would exert its neuroprotective neurons after peripheral axotomy, since it is known to reduce the oxidative damage in a variety of experimental neuropathologies, in which nitric oxide (NO) is involved [32]. Melatonin is believed to work via electron donation to directly detoxify free radicals such as the highly toxic hydroxyl radical [33]. The neuronal isoform of nitric oxide synthetase (nNOS), a NADPH-dependent diaphorase, is considered to play a role in motoneuron death induced by sciatic nerve transaction. Based on evidence, melatonin has strong antioxidant and cell-protective effects via mimicking the effects of calcium channel blockers [34]. Rogerio et al. have found that melatonin at doses of 1 to 50 mg/kg decreased neuronal death, whereas doses of 50 to 100 mg/kg caused failure to thrive, seizures, or death [35]. Turgut et al. have demonstrated the effect of melatonin in preventing neuroma formation of transacted sciatic nerve in rats. Melatonin enhanced axonal regeneration due to its inhibitory effect on neuroma formation [36]. Peripheral nerve injury that requires surgical repair does not result in complete recovery because of collagen of scar formation, ischemia, free oxygen radical damage, and other factors [34]. Although there are great numbers of studies that have mentioned protective effects of melatonin on peripheral nerve pathologies, there are also some studies that report toxic effects of melatonin on peripheral nerves [29].

### Effects of topically administered methylprednisolone on peripheral nerve regeneration

Methylprednisolone has been intensely investigated. Because of its pharmacological properties, it is considered to be neuroprotective. A primary neuroprotective mechanism of action in each of these cases is hypothesized to involve the ability of high doses of methylprednisolone to inhibit oxygen free radical-induced lipid peroxidation, although additional mechanisms may contribute [37]. Topically administered dexamethasone on peripheral nerve offers the benefit of cost savings as well as avoiding the complications associated with systemic administration. Dexamethasone loaded in silicone tube can improve functional recovery and morphometric indices of sciatic nerve [38]. Nachemson et al. have reported that methylprednisolone suppresses scar formation and improves axonal regeneration after transaction and suture of rat peripheral nerves [39]. Boa et al. have reported that methylprednisolone in early treated rats has reduced lipid peroxidation and inhibited arachidonic acid hydrolysis following spinal cord injury [40]. Suslu et al. have evaluated the effect of preoperatively locally administered dexamethasone on nerve recovery after induced nerve crush injury, and they concluded that local dexamethasone is more effective than systemic dexamethasone [41]. Dexamethasone and vitamin B12 are currently used in the clinic to treat peripheral nerve damage, but their mechanisms of action remain incompletely understood. Sun et al. concluded that dexamethasone and vitamin B12 promote peripheral nerve repair in a rat model of sciatic nerve injury through the upregulation of brain-derived neurotrophic factor (BDNF) of expression [42].

### Effects of topically administered vitamin B12 on peripheral nerve regeneration

B vitamins were reported to attenuate degenerating processes in the nervous system and therefore have been clinically administered in a combination of B_1_ (thiamine), B_6_ (pyridoxine), and B_12_ (cobalamine; Cbl) [43]. Vitamin B12 is a micronutrient that plays significant roles in numerous biological processes. B12 deficiency leads to methionine deficiency which required for the synthesis of both phospholipids and myelin. In addition, vitamin B12 has shown antioxidant properties [44]. Okada et al. showed that vitamin B12 provides a basic for more beneficial treatments of nervous disorders through effective systemic or local delivery of high doses of methylcobalamin to target organs [45]. Vitamin B12 is also a good scavenger of the reactive oxygen species and is suggested to be a good neuroprotectant. Hobenaghi et al. revealed that the regeneration and healing in dorsal spinal ganglion will be improved by increase of administration time and vitamin B12 dose, indicating that such vitamin was able to progress recovery process of peripheral nerve damage in experimental rats [46].

## Methods used for evaluating the success of the regenerative process after nerve repair in experimental research

There are several methods for evaluating peripheral nerve regeneration after nerve repair: morphologic, electrophysiological, biochemical, and functional methods. These methods can be performed with a large number of anatomic techniques that can provide new insights into the process of peripheral nerve regeneration [47].

### Morphological method

Morphological analysis is the very common method for the study of the peripheral nerve regeneration [48]. The morphometry method serves to describe structures in quantitative terms and in particular reveals minimal morphological differences between states of function [49]. Quantitative estimation of nerve fiber morphology and functional assessment is a very important tool in nerve regeneration research. Raimondo et al. mentioned eight geometrical parameters that can be used for the assessment of nerve fibers, which are number of fibers, density of fibers, diameter of fibers and axon, myelin thickness, myelin thickness/ratio and fiber diameter/axon diameter ratio or axon diameter, and fiber diameter/axon diameter ratio or axon diameter/fiber diameter (g-ratio)[50]. It is known that g-ratio equals 0.6, which approximates average values observed in most nerves; the relationships are theoretically optimal to the spread of current on the node of Ranvier to the next [51]. Count and measure of myelinated fibers can be performed with manual or current digital technique (automatic) [52]. It is well known that manual morphometry is difficult to perform and requires more time, whereas fully automatic computerized image analysis system is more precise and far less time consuming. Histograms of myelinated fiber size and median fiber diameter provide quantitative date on the regenerative process. Through electron microscopic photographs, we can obtain a detailed view of cell organelles and laminar structure of myelin and small unmyelinated fibers. Immunochemistry method can be used to evaluate axon regeneration in the early phase after injury [53]. According to Santos et al., the quantitative parameters of nerve fibers can vary significantly depending on the nerve level and on the distance from the point of lesion [54]. Therefore, the average values obtained from measurements made in different segments of the nerve should be calculated. Studies suggest that there is no single morphological technique which can be considered superior for the evaluation of peripheral nerve regeneration [55]. However, it is important to choose the appropriate methods for specific examinations in the context of examining the structure of nerve. Also, it is important to consider the cost benefit ratio for the selected method. Histomorphometry is often the final step of morphological investigation. Although apparently, simple quantitative morphology is tricky and should be carried out carefully in order to avoid the bias creeps in to the estimates [56]. Although there is a positive correlation between morphological and functional parameters of nerve regeneration, occasionally, this correlation is absent or is not satisfactory.

### Electrophysiological evaluation

For the assessment of the regeneration and functional restitution after nerve injury, electrophysiological tests should be applied. These tests are commonly used in clinical practice and can also be performed in animal models. Electrophysiological tests provide a quantitative measurement in the normal and pathological state. Neurological studies with the peripheral nerve injuries involved and integrated approach using sensor and motor nerve conduction studies and electromyography (EMG), as well as motor- and sensory-evoked potentials in some instances, as helpful adjuncts to the clinical assessment [57]. These tests are commonly used in clinical practice and can be also performed in animal models to determine the nature of the disorders and their severity. EMG serves to evaluate the motor unit action potentials (MUAPs) [58].

Nerve action potential (NAP) recording is a proven and useful tool in the surgical management of nerve injury [59]. The evoked action potential of individual axons has a characteristic spike waveform and conduction velocity (CV), which are dependent on the type of nerve fiber (diameter and myelination) and physiological state (temperature, metabolism) [60]. Two extracellular recording electrodes pick up the maximally evoked electrical activity of all active fibers, giving a summed potential called a compound nerve action potential (CNAP) [53]. CV and amplitude of compound muscle action potential (CMAP) are used as indicators for neural regeneration [61]. CMAP represents the summation of the action potentials of all the excited muscle fibers that respond to the nerve stimulation. Therefore, motor nerve conduction tests provide selective information on the function and regeneration of α-motor axons [58]. Each wave of CV can be calculated by measuring the latency of the distance between stimulating and recording electrode. In regenerative nerves, the CV is altered by changes in axon diameter and myelination and remains below normal value for a long time after successful regeneration. The CNAP amplitude does not correlate with a total number of regeneration myelinated fibers, but has a highly significant relation following an exponential function, which is the number of large myelinated fibers [62].

The latest data from the literature show that ENG is not often used for evaluation of neural regeneration because it is an invasive method. However, it is suitable for longitudinal measurements using appropriate recording device. Neurophysiological measurements *ex vivo* enable higher reproducibility and signal quantification as compared to *in vivo* measurements due to several factors that can change, such as distance, temperature, etc. The degree of the damaged nerve can be estimated by comparing the electrophysiological values between the damaged nerve and the respective contralateral nerve.

### Motor nerve conduction tests

According Fullerton and Barnes 1966, motor nerve conduction is a widely used electrophysiological technique, which serves for assessment of nerve regeneration and neuropathies in experimental models since in the 1960s [63]. These tests have a larger amplitude potential than direct nerve conduction tests. The amplitude of the CMAP is determined by the number of muscle fibers innervated. In contrast to the CNAP, the CMAP amplitude is little affected by the distance of the conduction. The motor CV can be calculated in the nerve segment between two stimulation sites by dividing the difference of the latencies by the distance between the stimulation points. The CMAP amplitude is proportional to the number of motor axons regenerated and the side for the corresponding motor units in the recorded muscle [58]. It is known that complete recovery of the amplitude does not show that all axons in the injured nerve are regenerated.

### Functional evaluation of the nerve repair

There are several tests for functional evaluation of the nerve repair. In aadition to the large variety of electrophysiological and morphological tests for studying nerve regeneration in animals, there are several other tests for the functional evaluation of nerve repair. One of the best known tests is the sciatic function index (SFI) in rats. For rabbits, the toe-spreading reflex has been suggested as a valuable index for peroneal function [64]. Many experiments on nerve regeneration are performed in recent years using SFI as a measure of functional loss. According de Medinaceli et al. (1982), there are a wide variety of techniques that can be used to study the recovery of the peripheral nerve injury, but it should be taken into consideration that unlike in, humans these techniques are difficult to perform in animals [65]. Nerve regeneration in animal experiments can be assessed with histomorphometry and electrophysiology measurements; however, these methods do not always correlate with recovery motor and sensory functions [66]. It is recommended to use various methods for overall assessment of nerve function [67]. One of these methods is a walking track which was designed to visualize and record gait of rats. Rats were allowed to walk freely in a walkway in order to analyze their visible footprints by stepping in developer on X-ray film or paint on paper [65]. Bervar introduced a new method that can perform credible and fast footprint analysis [68]. For evaluation of the footprints, three different parameters are used: print length (PL), toe spread (TS), and intermediary toe spread (ITS) [69].

## Conclusion

Traumatic injuries of the peripheral nerves are very common. Surgical repair of the damaged nerve is often complicated by scar tissue formation around the damaged nerve. Numerous data from literature suggest that in order to prevent scar formation, topical pharmacological agents at the site of peripheral nerve repair can be applied. In this review paper, we presented some of the most commonly used pharmacological agents in animal models. These pharmacological agents prevent scar formation at the site of peripheral nerve repair and accelerate nerve regeneration. The mechanisms of action by which these substances operate in peripheral nerve regeneration are described in detail. There are several methods for nerve repair evaluation following topical application of different pharmacological agents, such as morphometry, electrophysiology methods, and walking track analysis. These methods are of great value in functional assessment of the damaged nerve. In summary, topical pharmacological agents have a notable effect in the prevention of scar tissue formation and contribute markedly in nerve regeneration. It is understandable, however, that complete regeneration and functional recovery will almost never be achieved.
